# Serum Glycan Markers for Evaluation of Disease Activity and Prediction of Clinical Course in Patients with Ulcerative Colitis

**DOI:** 10.1371/journal.pone.0074861

**Published:** 2013-10-07

**Authors:** Koji Miyahara, Kazuhiro Nouso, Shunsuke Saito, Sakiko Hiraoka, Keita Harada, Sakuma Takahashi, Yuki Morimoto, Sayo Kobayashi, Fusao Ikeda, Yasuhiro Miyake, Hidenori Shiraha, Akinobu Takaki, Hiroyuki Okada, Maho Amano, Kazuko Hirose, Shin-Ichiro Nishimura, Kazuhide Yamamoto

**Affiliations:** 1 Department of Gastroenterology & Hepatology, Okayama University Graduate School of Medicine, Dentistry, and Pharmaceutical Sciences, Okayama, Okayama, Japan; 2 Department of Molecular Hepatology, Okayama University Graduate School of Medicine, Dentistry, and Pharmaceutical Sciences, Okayama, Okayama, Japan; 3 Department of Endoscopy, Okayama University Hospital, Okayama, Okayama, Japan; 4 Field of Drug Discovery Research, Faculty of Advanced Life Science & Graduate School of Life Science, Hokkaido University, Sapporo, Hokkaido, Japan; 5 Medicinal Chemistry Pharmaceuticals, Co., Ltd., Sapporo, Hokkaido, Japan; Osaka University Graduate School of Medicine, Japan

## Abstract

**Background:**

The aims of this study were to determine the change of whole-serum *N*-glycan profile in ulcerative colitis (UC) patients and to investigate its clinical utility.

**Methods:**

We collected serum from 75 UC patients at the time of admission and the same number of age/sex-matched healthy volunteers. Serum glycan profile was measured by comprehensive quantitative high-throughput glycome analysis and was compared with disease activity and prognosis.

**Results:**

Out of 61 glycans detected, 24 were differentially expressed in UC patients. Pathway analysis demonstrated that highly sialylated multi-branched glycans and agalactosyl bi-antennary glycans were elevated in UC patients; in addition, the glycan ratio *m/z* 2378/1914, which also increased in UC, showed the highest Area under Receiver Operating Characteristic curve (0.923) for the diagnosis of UC. Highly sialylated multi-branched glycans and the glycan ratio *m/z* 2378/1914 were higher in the patients with total colitis, Clinical Activity Index >10, Mayo endoscopic score 3, or a steroid-refractory status. In particular, the glycan ratio *m/z* 2378/1914 (above median) was an independent prognostic factor for the need for an operation (hazard ratio, 2.67; 95% confidence interval, 1.04–7.84).

**Conclusions:**

Whole-serum glycan profiles revealed that the glycan ratio *m/z* 2378/1914 and highly sialylated multi-branched glycans increase in UC patients, and are correlated with disease activity. The glycan ratio *m/z* 2378/1914 was an independent predictive factor of the prognosis of UC.

## Introduction

Ulcerative colitis (UC) is a chronic intestinal disorder of unknown etiology with a typically relapsing and remitting course [1]. The evaluation of disease activity is crucial for selecting the therapy and predicting the clinical course, so various laboratory biomarkers [2], clinical indices [3], and endoscopic evaluations [4] have been used simultaneously to determine the activity. An ideal marker is minimally invasive and can be used to monitor the disease activity or predict the clinical course objectively; however, existing clinical indices often use variables subjectively assessed by the patient or a physician, and require endoscopic evaluations that are sometimes invasive and not suitable for routine use.

C-reactive protein (CRP) is one of the most useful markers correlated significantly with the disease activity of UC, and white blood cell count (WBC), platelet, erythrocyte sedimentation rate (ESR) and albumin are also used frequently [2], [5], [6]. However, CRP was reported to correlate less well with disease activity in UC than in Crohn's disease (CD) [2], [7], [8]. In addition, there was concern that some of the existing biomarkers are influenced by disease-modifying drugs and corticosteroids [2], [9], although the influence on CRP was still unclear [5].

Serum glycans have been reported to be promising diagnostic markers for chronic inflammatory disease, including inflammatory bowel disease (IBD) [10], [11]. However, the majority of reported studies investigated glycans attached to a particular protein, such as acute phase proteins or immunoglobulin (Ig) G; therefore, the full picture of the alteration of glycan profile in patients with UC has not been elucidated.

A new technology for glycan-specific enrichment, the “glycoblotting method,” was recently developed [12]. This method enables comprehensive analysis of serum glycans and can achieve high-throughput and quantitative glycomics. The aims of this study were to determine whole glycan expressions in patients with UC using this method and to evaluate the potential use of glycan profiles as new clinical markers for the evaluation of disease activity and for prediction of the clinical course of UC.

## Materials and Methods

### Patients

Seventy-five patients with UC who were admitted to Okayama University Hospital between January 1997 and December 2007 and the same number of healthy volunteers (HLT) that matched the patients in terms of age and sex were enrolled in this study. We also enrolled 31 CD patients and compared glycan profiles in the patients and that in age sex-matched HLT controls. Seventy-one of UC and 28 of CD patients had already received therapies before admission using aminosalicylate, corticosteroids, mercaptopurine/azathioprine, cyclosporine, leukocytapheresis, and/or elemental diet. The profiles of the patients upon admission and the HLT are shown in Table 1. Median disease duration, defined as the period between the time of onset and the start of the first treatment upon admission during the study period, was 3 and 9 years in UC and CD patients, respectively. Median clinical activity index (CAI) in UC patients was 10, which was composed of seven variables: number of stools, blood in stools, investigator's global assessment of symptomatic state, abdominal pain or cramps, temperature due to colitis, extraintestinal manifestations, and laboratory findings [13]. Sixteen patients with UC were in symptomatic remission (CAI score <5), who were admitted to have examinations with colonoscopy.

**Table 1:** Characteristics of patients with ulcerative colitis and Crohn's disease on admission and healthy volunteers.

Variables	UC (n = 75)	CD (n = 31)	HLT (n = 75)
Age, yr, median (IQR)	38 (30–54)	34 (25–39)	37 (30–50)
Male/female	42/33	19/12	42/33
Disease duration, yr, median (IQR)	3 (1–9)	9 (3–16)	-
Disease location, n			
Total colitis/left-sided colitis	56/19	-	-
Small bowel/colon/both	-	10/7/14	-
CAI score, median (IQR)	10 (5–13)	-	-
CDAI score, median (IQR)	-	138 (101–172)	-
Mayo endoscopic score, n, 0/1/2/3	0/15/20/40	-	-
Laboratory data, median (IQR)			
WBC [×10^3^/mm^3^]	8.5 (6.1–11.3)	5.6 (4.3–7.9)	-
PLT [×10^4^/mm^3^]	33 (26–45)	38 (26–44)	-
ESR [mm/hr]	36 (14–67)	31 (19–55)	-
ALB [g/dL]	3.2 (2.6–3.8)	3.5 (3.1–4.0)	-
CRP [mg/dL]	0.8 (0.2–4.8)	0.5 (0.1–2.4)	-
Treatment before admission, n (%)			
Aminosalicylate	67 (89)	24 (77)	-
Corticosteroids	52 (69)	2 (6)	-
Mercaptopurine/azathioprine	13 (17)	6 (19)	-
Cyclosporine	3 (4)	0 (0)	-
Leukocytapheresis	20 (27)	0 (0)	-
Elemental diet	0 (0)	19 (61)	-

Abbreviations: ALB, albumin; CAI, clinical activity index; CD, Crohn's disease; CDAI, Crohn's disease activity index; CRP, C-reactive protein; ESR, erythrocyte sedimentation rate; HLT, healthy volunteers; IQR, interquartile range; PLT, platelet; UC, ulcerative colitis; WBC, white blood cell count.

Median Crohn's disease activity index (CDAI) in CD patients was 138, which was composed of eight variables: number of liquid or very soft stools, abdominal pain score in one week, general well-being, sum of physical findings per week, antidiarrheal use, abdominal mass, the values of hematocrit, and percentage deviation from standard weight [14].

### Diagnosis and assessment of disease

The diagnosis of UC was based on conventional criteria [15]. Patients without a definite diagnosis of UC or CD (e.g. indeterminate colitis) were excluded. Disease severity of UC was assessed using the extent of disease confirmed by endoscopy, CAI score, and the endoscopic component of the Mayo Scoring System for assessment of UC activity (Mayo endoscopic score): Score 0 =  normal, endoscopic remission; Score 1 =  mild, erythema, decreased vascular pattern, mild friability; Score 2 =  moderate, marked erythema, absent vascular pattern, friability, erosions; and Score 3 =  severe, spontaneous bleeding, ulceration [16]. Steroid-refractoriness was defined as little or no improvement after two weeks of systemic steroid therapy (at least 30 mg of prednisolone daily) as previously reported [17].

### Glycoblotting

We collected serum from all the patients at the time of admission, and from the HLT. The blood samples were centrifuged for 10 minutes at 15,000×*g*, and supernatant were frozen immediately and stored below −30°C until use. Glycoblotting was performed according to a procedure described previously [12]. Briefly, 10μL serum samples were applied to an automated machine, “Sweetblot” prototype 7 (System Instruments Co.), for pre-treatment and for glycoblotting. After enzymatic cleavage from proteins, glycans were captured on BlotGlyco H beads (Sumitomo Bakelite, Co.), and sialic acids were methyl-esterified. The processed glycans were tagged with benzyloxyamine (BOA) and released from the beads, followed by detection by MALDI-TOF-MS (Ultraflex 3, Bruker, Germany).

### Statistical analysis

Student's t-test was used to compare the continuous data. The clinical course of undergoing operation was compared using the Kaplan-Meier method and evaluated by log-rank test. Cox's proportional hazards model was used to analyze hazard ratios (HR). The JMP (version 8) software package (SAS Institute, Cary, North Carolina, USA) was used for the analyses and p<0.05 was considered significant. Bonferroni correction was used for multiple comparisons and p<0.05/61 was considered significant for evaluation of the difference between the expression in the UC patients and that in the HLT. Pathway analysis was conducted using the Cytoscape program (www.cytoscape.org). To assess the statistical accuracy, validation was implemented by bootstrap methods (10,000 re-sampling) using SAS (version 9.3) software package (SAS Institute, Cary, North Carolina, USA).

### Ethics Statement

Written informed consent for taking the serum and using clinical data was obtained from all the patients and the HLT. The study protocol conformed to the ethical guidelines of the World Medical Association Declaration of Helsinki and was approved by the Okayama University Ethics Committee (authorization number #669).

## Results

### Changes in serum glycan profiles

**Figure 1:**
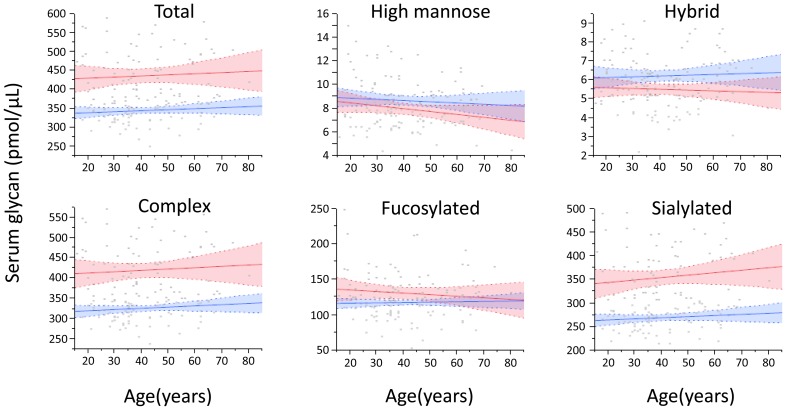
Serum levels of glycans with different structures. The total glycan level in the serum of ulcerative colitis patients (UC, *red*) was significantly higher than that of healthy controls (HLT, *blue*), regardless of age. In particular, complex type and sialylated glycans were significantly increased in UC, although the expression of high-mannose type, hybrid type, or fucosylated glycans did not differ between UC and HLT. Lines and colored areas indicate regression curves and 95% CI, respectively.

Glycome analysis by Sweetblot revealed the expression of 61 BOA-labeled glycans with a molecular weight (*m/z*) ranging from 1362.481 to 4157.522. Five glycans were classified as high-mannose type, six as hybrid type, and 50 as complex type (of these, 25 were bi-antennary, 10 were tri-antennary, and 15 were tetra-antennary). Forty-three glycans were sialylated and 33 contained a fucose moiety. The total glycan level in the serum of UC patients was significantly higher than that of healthy controls regardless of age (Figure 1). In particular, complex type and sialylated glycans were significantly increased in UC, although the expression of high-mannose type, hybrid type, or fucosylated glycans did not differ between UC and HLT.

From 10,000 bootstrap re-sampling, the elevation of complex type and sialylated glycans in UC patients were confirmed. Point estimations of mean values of complex type glycans were 422 pmol/μL in UC and 327 pmol/μL in HLT (p<0.0001), and those of sialylated glycans were 355 pmol/μL in UC and 270 pmol/μL in HLT (p<0.0001).

### Pathway analysis

**Figure 2:**
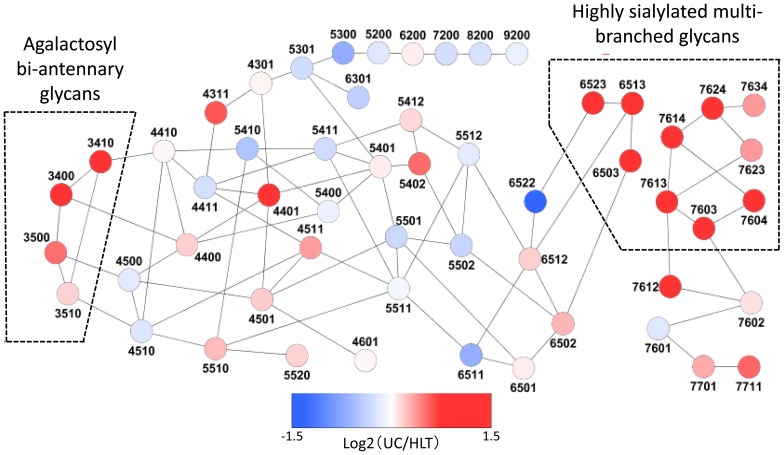
Pathway analysis of glycan biosynthesis. Relative glycan expression is indicated by *circle color* according to log2(patient/HLT) value. Color mapping is shown in the color bar. The *four-digit* numbers indicate the numbers of hexose (galactose and mannose), N-acetyl- hexosamine (N-acetyl glucosamine), fucose, and sialic acid moieties, in this order. All the samples used in this analysis were obtained from mean values of patients or healthy controls. Increased biosynthesis of multi-branched glycans, especially highly sialylated (≥3) glycans, and agalactosyl bi-antennary glycans was clearly observed in ulcerative colitis patients. Glycans *m/z* 2378 and 1914 correspond to 5402 and 5410 in this figure, respectively.

Pathway analysis was performed to reveal trends in glycan biosynthesis in UC patients (Figure 2). All the samples used in this analysis were obtained from mean values in UC or HLT. Increased biosynthesis of multi-branched (tri- and tetra-antennary) glycans, especially highly sialylated (≥3) glycans, and agalactosyl bi-antennary glycans, which lacked terminal galactose, was clearly observed in UC patients.

### Glycans to discriminate between UC and HLT

Among the 61 glycans examined, the expression of 24 glycans differed significantly between UC patients and HLT after Bonferroni correction (Table S1). When we assessed the utility of these glycans for discriminating UC from HLT, glycan *m/z* 2378 showed the highest Area under Receiver Operating Characteristic curve (AUROC; 0.911) in 14 up-regulated glycans, while glycan *m/z* 1914 did so in 10 down-regulated glycans (AUROC; 0.844). The ratio of these glycans (*m/z* 2378/1914) was significantly higher in UC (median, 22.4; interquartile range, 15.9–28.5) than in HLT (median, 10.5; interquartile range, 8.5–12.0; p<0.0001, Student's t-test), and showed a higher AUROC (0.923) than any single glycan.

### Correlation between glycans and clinical manifestations in UC patients

**Figure 3:**
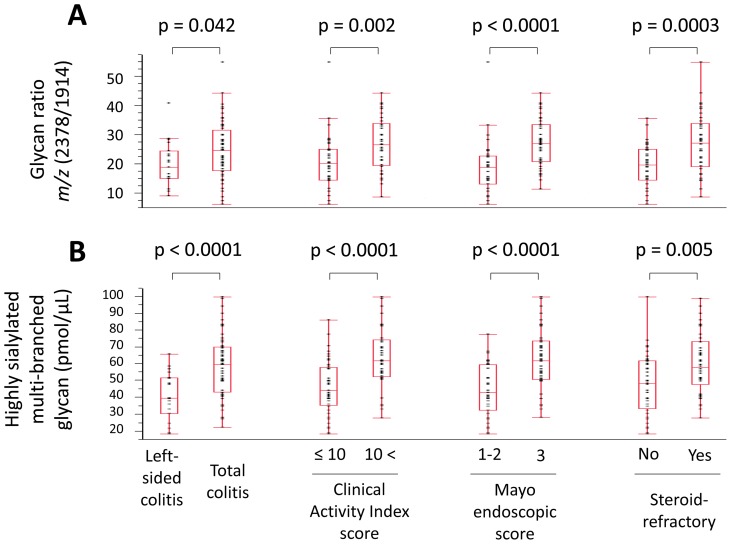
Differentially expressed serum glycans with characteristics of ulcerative colitis. Box-plot of (A) the glycan ratio *m/z* 2378/1914 and (B) highly sialylated multi-branched glycans, which were significantly higher in the patients with total colitis, Clinical Activity Index >10, Mayo endoscopic score 3, or a steroid-refractory status, compared with left-sided colitis, Clinical Activity Index ≤10, Mayo endoscopic score 1–2, or no steroid-refractory status, respectively.

To investigate whether the glycans that increased in UC patients correlated with clinical manifestations of UC, we compared the expression of agalactosyl bi-antennary glycans, highly sialylated (≥3) multi-branched glycans, and the glycan ratio *m/z* 2378/1914 in association with extent of disease, CAI score, Mayo endoscopic score, and steroid-refractory status (Figure 3). The values of agalactosyl bi-antennary glycans and highly sialylated multi-branched glycans were calculated by summing for four and 13 glycans, respectively. The glycan ratio *m/z* 2378/1914 (Figure 3A) and the level of highly sialylated multi-branched glycans (Figure 3B) were significantly higher in the patients with total colitis, CAI >10, Mayo endoscopic score 3, or a steroid-refractory status, compared with those with left-sided colitis, CAI ≤10, Mayo endoscopic score 1–2, or no steroid-refractory status, respectively. On the other hand, the expression of agalactosyl bi-antennary glycans did not differ in association with extent of disease, CAI score, Mayo endoscopic score, and steroid-refractory status.

From 10,000 bootstrap re-sampling, the difference of highly sialylated multi-branched glycans were confirmed. Point estimations of mean values between each clinical manifestations were as follows: left-sided colitis (40.8 pmol/μL) vs. total colitis (58.7 pmol/μL, p<0.0001), CAI score ≤10 (46.4 pmol/μL) vs. >10 (63.6 pmol/μL, p<0.0001), Mayo endoscopic score 1–2 (44.4 pmol/μL) vs. 3 (62.8 pmol/μL, p<0.0001), and steroid-refractory ‘No’ (48.0 pmol/μL) vs. ‘Yes’ (60.2 pmol/μL, p = 0.001). Those of the glycan ratio *m/z* 2378/1914 were as follows: left-sided colitis (20.0) vs. total colitis (24.7, p = 0.016), CAI score ≤10 (20.5) vs. >10 (27.2, p<0.0001), Mayo endoscopic score 1–2 (19.0) vs. 3 (27.4, p<0.0001), and steroid-refractory ‘No’ (19.6) vs. ‘Yes’ (27.3, p<0.0001).

### The utility of glycan markers for predicting disease prognosis

**Figure 4:**
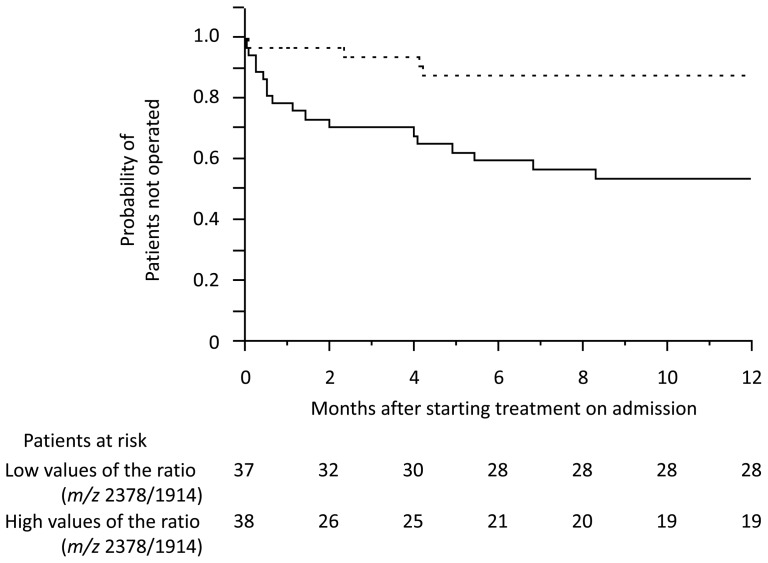
Clinical course of requiring surgery in patients with high and low values of the glycan ratio *m/z* 2378/1914. Kaplan-Meier plot of the patients with high (solid line) and low (dotted line) values of the glycan ratio *m/z* 2378/1914 (cut-off: median). Time to operation was significantly shorter in patients with high values than in those with low values of the glycan ratio *m/z* 2378/1914 (p = 0.0006; log-rank test).

To evaluate the utility of glycan markers for predicting disease prognosis, we compared the rate of proctocolectomy between the patients with high and low values of glycan ratio *m/z* 2378/1914 and highly sialylated multi-branched glycans, as well as existing biomarkers: WBC, platelet, ESR, albumin, and CRP. The patients with high values of glycan ratio *m/z* 2378/1914 showed a shorter time to operation than those with low values (cut-off, median; p = 0.0006: Figure 4). The HR (above the median) for requiring an operation was higher for glycan ratio *m/z* 2378/1914 (HR, 4.33; 95% confidence interval (CI), 1.85–11.8), as well as CAI, >10 (HR, 4.13; 95% CI, 1.86–10.0) and Mayo endoscopic score, 3 (HR, 5.10; 95% CI, 2.19–13.9) than for the highly sialylated multi-branched glycans (HR, 3.04; 95% CI, 1.37–7.39), CRP (HR, 2.47; 95%CI, 1.13–5.79), or other existing biomarkers in univariate analysis (Table 2). In addition, the glycan ratio *m/z* 2378/1914 was an independent prognostic factor for requiring an operation in multivariate analysis, when only variables that demonstrated a p-value <0.1 in univariate analysis were entered into the multiple logistic regression model.

**Table 2:** Prognostic factors for requiring an operation in patients with ulcerative colitis.

Variables	Univariate analysis			Multivariate analysis		
	HR	95% CI	p-values	HR	95% CI	p-values
Extent of disease (Total colitis)	1.85	0.76–5.51	0.184			
CAI score (>10)	4.13	1.86–10.0	<0.001	2.30	0.93–6.15	0.069
Mayo endoscopic score (3)	5.10	2.19–13.9	<0.001	3.09	1.17–9.12	0.021
WBC (> median)	0.71	0.33–1.50	0.369			
PLT (> median)	0.92	0.43–1.94	0.830			
ESR (> median)†	1.51	0.66–3.53	0.328			
ALB (> median)	0.49	0.22–1.04	0.064	1.34	0.55–3.09	0.511
CRP (> median)	2.47	1.13–5.79	0.023	1.29	0.54–3.29	0.573
Glycan ratio *m/z* 2378/1914 (> median)	4.33	1.85–11.8	<0.001	2.67	1.04–7.84	0.040
Highly sialylated multi-branched glycans (> median)	3.04	1.37–7.39	0.006			

Note: Only variables that demonstrated a p-value <0.1 in univariate analysis were entered into the multiple logistic regression model. Highly sialylated multi-branched glycans was excluded in multivariate analysis to avoid the multicollinearity with Glycan ratio *m/z* 2378/1914.

† The data of ESR were not available in 13 patients.

Abbreviations: ALB, albumin; CAI, clinical activity index; CRP, C-reactive protein; ESR, erythrocyte sedimentation rate; PLT, platelet; WBC, white blood cell count.

### Glycan profiles in CD

We detected 18 glycans (15 up-regulated and 3 down-regulated) that were differentially expressed between CD patients and HLT (Table S2). The expression profile in CD was similar to that in UC, and eleven out of the 18 glycans were the same. In addition, glycan *m/z* 2378 (AUROC; 0.948) and *m/z* 1914 (AUROC; 0.879) showed the highest AUROC in 15 up-regulated and in 3 down-regulated glycans, respectively.

## Discussion

This study first analyzed whole-serum *N*-glycan profiles in UC patients using Sweetblot, a high-throughput and automated glycomics system that can measure glycans comprehensively and quantitatively. The total amount of *N*-glycans was elevated in UC patients, and pathway analysis demonstrated that the increased biosynthesis was remarkable for highly sialylated multi-branched glycans and agalactosyl bi-antennary glycans. In addition, we also found that a particular glycan ratio (*m/z* 2378/1914) was significantly higher in UC than in HLT. Serum expression of highly sialylated multi-branched glycans and the glycan ratio *m/z* 2378/1914 were well correlated with clinical manifestations of UC, such as the extent of disease, CAI score, Mayo endoscopic score, and steroid-refractory status, and the results were validated by using bootstrap method. Furthermore, the expression of the glycan ratio *m/z* 2378/1914 was an independent prognostic factor for requiring an operation. The expression of glycans in UC was similar to that in CD.

Glycans with multiple branches, sialylation, and a consequently induced sialyl Lewis X (SLe^x^) epitope, which involves the structure of outer branch fucosylation and sialylation [18] and is related to leukocyte-endothelial adhesion [19], were reported to increase in various chronic inflammatory diseases, including rheumatoid arthritis [20], Crohn's disease [21], and cancers [21]–[23]. These alterations of glycan were shown on acute-phase proteins such as haptoglobin [20]–[22] and alpha 1-acid glycoprotein [24]–[26], and were considered to be common features of long-term inflammation [10]. However, SLe^x^ epitope was observed in only two glycans (*m/z* 3341 and 4011) among the 24 glycans in our study examining UC patients. This might have been because the SLe^x^ epitope was down-regulated by prior therapies including corticosteroids [27]. On the other hand, the glycan *m/z* 2378, which is a fully sialylated bi-antennary glycan, showed a higher AUROC for the diagnosis of UC than all other glycans with multiple branches and/or SLe^x^ epitope. Interestingly, this glycan retained its correlation with CAI score even in patients treated with a high dose of corticosteroids (≥50 mg of prednisolone daily), whereas many other glycans or existing biomarkers did not (data not shown). This observation indicates that glycan *m/z* 2378 would be suitable as a biomarker of UC patients with prior therapies, and it is the best for predicting prognosis when divided by the value of glycan *m/z* 1914. In addition, out preliminary study with autoimmune hepatitis and autoimmune pancreatitis showed no elevation of the ratio (*m/z* 2378/1914) so that the elevation might be specific phenomenon in UC and CD patients (data not shown).

Alteration of *N*-linked glycans attached to IgG was previously reported in UC patients. Shinzaki et al. reported that the serum levels of agalactosylated bi-antennary glycan-attached IgG increased in UC patients, and were correlated with disease activity or the extent of inflammation [11]. Our result supports this observation in terms of agalactosylated bi-antennary glycans increasing in UC, but they were not correlated with disease activity or the extent of inflammation in the present study. Agalactosylation of IgG is correlated with lowered galactosyltransferase (GTase) activity in B and T cells [28], and modulates immune function to enhance antibody-dependent phagocytosis in vitro [29], but galactosylation profiles of other glycoproteins have not been elucidated in chronic inflammatory conditions. Our analysis of whole glycans in serum showed that multiple branching and sialylated glycans increased according to the progression of disease activity or the extent of inflammation, resulting in abolition of the increase of agalactosylation with disease progression.

We demonstrated that the glycan ratio *m/z* 2378/1914 was an independent prognostic factor for requiring an operation when the cut-off value was set at median (sensitivity  = 66.0%, specificity  = 78.6%). By changing a cut-off value at the 25th percentile (16.0) in UC patients, clinical importance of the ratio increases. Only 2 out of 19 patients (10.5%) required surgery in low *m/z* 2378/1914 group so that the specificity with this setting is 92.9% and the sensitivity is 36.2%. The biological implications of the glycan *m/z* 1914 and *m/z* 2378 have not been elucidated; however, these glycans seem to correlate with inflammation. The glycan *m/z* 1914, which is a digalactosyl bi-antennary glycan, is mainly attached to IgG in serum [30]. Therefore, its decrease might represent agalactosylation of IgG-bound glycans, which is closely related with mucosal inflammation [31]. On the other hand, the glycan *m/z* 2378, which is the most abundant glycan in serum, mainly attached to liver secreted glycoproteins [30]; so the increase of the glycan would reflect the up-regulation of various glycoproteins on inflammatory conditions.

Although we successfully revealed glycan profiles in UC patients, there are several limitations to this study. Firstly, it involved retrospective analysis with a small sample size, and glycans were measured at only one point, so we did not evaluate the change of glycan profiles over the clinical course. Secondly, we did not analyze the serum of patients with intestinal inflammation unrelated to IBD, or validate the results in another set of UC patients. Another limitation is that the majority of patients (71 out of 75) had already received therapies before admission, which might have influenced the serum levels of glycans upon admission. In addition, we could not clarify whether alterations of serum glycan were caused by alteration of carrier protein profile or by modification of attached glycans by this method. Further examination to find out ligands of each glycan and the biological effects of glycan alterations are necessary.

Despite these limitations, we first demonstrated whole-serum glycan profiles in UC patients and clearly demonstrated that the glycan ratio *m/z* 2378/1914 and highly sialylated multi-branched glycans increase in UC patients, and are correlated with disease activity. In addition, the glycan ratio *m/z* 2378/1914 was the best predictive factor for the prognosis of UC among the markers measured in this study. However, further prospective and longitudinal studies are needed to confirm these results.

## Supporting Information

Table S1 **Differentially expressed serum glycans in patients with ulcerative colitis.**
(DOCX)

Table S2 **Differentially expressed serum glycans in patients with Crohn's disease.**
(DOCX)
