# Supplementary material for: Serum Glycan Markers for Evaluation of Disease Activity and Prediction of Clinical Course in Patients with Ulcerative Colitis
Source: PLoS One. 2013 Oct 7;8(10):e74861. doi: 10.1371/journal.pone.0074861 (PMC3792068; doi:10.1371/journal.pone.0074861)
Supplement: Table S1 — Differentially expressed serum glycans in patients with ulcerative colitis. (DOCX) [file pone.0074861.s001.docx]

**Table S1.** Differentially expressed serum glycans in patients with ulcerative colitis

| *Glycans* | | | Glycan expression | | | | |
| --- | --- | --- | --- | --- | --- | --- | --- |
| Code # | *m/z* | Monosaccharide composition† | UC, median (pmol/μL) | HLT, median (pmol/μL) | p-values | AUROC | Up/down regulated in UC |
| *High-mannose type* | | |  |  |  |  |  |
| #7200 | 1686 | 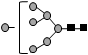 | 0.79 | 1.01 | 0.0001 | 0.728 | Down-regulated |
| #8200 | 1848 | 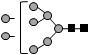 | 1.18 | 1.41 | 0.0004 | 0.682 | Down-regulated |
| *Hybrid type* | | |  |  |  |  |  |
| #5300 | 1565 | 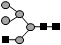 | 0.18 | 0.27 | < 0.0001 | 0.815 | Down-regulated |
| #5301 | 1870 | 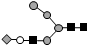 | 1.94 | 2.36 | < 0.0001 | 0.813 | Down-regulated |
| #6301 | 2032 | 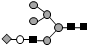 | 0.96 | 1.29 | < 0.0001 | 0.816 | Down-regulated |
| *Bi-antennary glycans of complex type* | | |  |  |  |  |  |
| #3400 | 1444 | 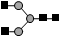 | 0.35 | 0 | < 0.0001 | 0.707 | Up-regulated |
| #3410 | 1590 | 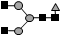 | 12.8 | 7.78 | < 0.0001 | 0.818 | Up-regulated |
| #3500 | 1647 | 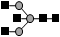 | 0.79 | 0.68 | < 0.0001 | 0.631 | Up-regulated |
| #5410 | 1914 | 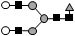 | 9.25 | 13.8 | < 0.0001 | 0.844 | Down-regulated |
| #4411 | 2057 | 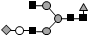 | 1.69 | 2.13 | 0.0003 | 0.668 | Down-regulated |
| #5411 | 2219 | 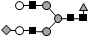 | 14.6 | 17.3 | < 0.0001 | 0.737 | Down-regulated |
| #5501 | 2276 | 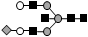 | 1.99 | 2.73 | < 0.0001 | 0.722 | Down-regulated |
| #5402 | 2378 | 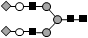 | 198 | 140 | < 0.0001 | 0.911 | Up-regulated |
| *Tri-antennary glycans of complex type* | | |  |  |  |  |  |
| #6600 | 2336 | 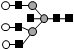 | 4.88 | 4.01 | 0.0001 | 0.665 | Up-regulated |
| #6511 | 2584 | 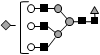 | 0.49 | 0.75 | < 0.0001 | 0.811 | Down-regulated |
| #6503 | 3049 | 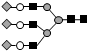 | 33.0 | 20.5 | < 0.0001 | 0.858 | Up-regulated |
| #6513 | 3195 | 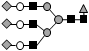 | 13.5 | 5.79 | < 0.0001 | 0.797 | Up-regulated |
| #6523 | 3341 | 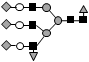 | 0.16 | 0.09 | < 0.0001 | 0.650 | Up-regulated |
| *Tetra-antennary glycans of complex type* | | |  |  |  |  |  |
| #7711 | 3153 | 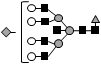 | 0.25 | 0.18 | 0.0005 | 0.654 | Up-regulated |
| #7603 | 3414 | 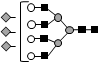 | 0.83 | 0.58 | < 0.0001 | 0.651 | Up-regulated |
| #7613 | 3560 | 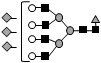 | 0.16 | 0.10 | < 0.0001 | 0.635 | Up-regulated |
| #7604 | 3719 | 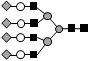 | 1.48 | 0.54 | < 0.0001 | 0.782 | Up-regulated |
| #7614 | 3865 | 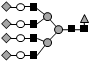 | 0.36 | 0.13 | < 0.0001 | 0.732 | Up-regulated |
| #7624 | 4011 | 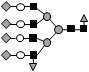 | 0.02 | 0 | < 0.0001 | 0.698 | Up-regulated |

Note: p-values are from Student’s t-test. Glycans with p < 0.05/61 are shown. # indicates the numbers of monosaccharides, the same as in Figure 2.

^†^Monosaccharide composition: *Rhombus*, sialic acid; *triangle*, fucose; *square*, *N*-acetyl glucosamine; *open circle*, galactose; *closed circle*, mannose.

Abbreviations: AUROC, Area under Receiver Operating Characteristic curve; HLT, healthy volunteers; UC, ulcerative colitis.
