# Supplementary material for: Serum Glycan Markers for Evaluation of Disease Activity and Prediction of Clinical Course in Patients with Ulcerative Colitis
Source: PLoS One. 2013 Oct 7;8(10):e74861. doi: 10.1371/journal.pone.0074861 (PMC3792068; doi:10.1371/journal.pone.0074861)
Supplement: Table S2 — Differentially expressed serum glycans in patients with Crohn's disease. (DOCX) [file pone.0074861.s002.docx]

**Table S2.** Differentially expressed serum glycans in patients with Crohn’s disease

| *Glycans* | | | Glycan expression | | | | |
| --- | --- | --- | --- | --- | --- | --- | --- |
| Code # | *m/z* | Monosaccharide composition† | CD, median (pmol/μL) | HLT, median (pmol/μL) | p-values | AUROC | Up/down regulated in CD |
| *Hybrid type* | | |  |  |  |  |  |
| #5300 | 1565 | 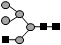 | 0.19 | 0.32 | < 0.0001 | 0.797 | Down-regulated |
| *Bi-antennary glycans of complex type* | | |  |  |  |  |  |
| #3410 | 1590 | 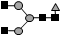 | 21.7 | 6.97 | < 0.0001 | 0.872 | Up-regulated |
| #3500 | 1647 | 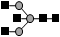 | 0.91 | 0.61 | < 0.0001 | 0.766 | Up-regulated |
| #5400 | 1768 | 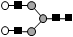 | 1.10 | 1.65 | 0.0003 | 0.766 | Down-regulated |
| #3510 | 1793 | 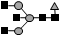 | 2.47 | 1.42 | 0.0001 | 0.771 | Up-regulated |
| #4401 | 1911 | 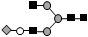 | 0.00 | 0.00 | 0.0005 | 0.692 | Up-regulated |
| #5410 | 1914 | 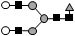 | 10.0 | 15.7 | < 0.0001 | 0.879 | Down-regulated |
| #5401 | 2073 | 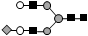 | 33.4 | 29.1 | 0.0003 | 0.745 | Up-regulated |
| #5402 | 2378 | 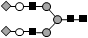 | 182 | 141 | < 0.0001 | 0.948 | Up-regulated |
| #5412 | 2524 | 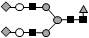 | 21.1 | 15.6 | < 0.0001 | 0.855 | Up-regulated |
| *Tri-antennary glycans of complex type* | | |  |  |  |  |  |
| #6512 | 2890 | 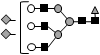 | 1.55 | 0.92 | 0.0003 | 0.754 | Up-regulated |
| #6612 | 2934 | 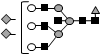 | 0.78 | 0.43 | 0.0003 | 0.744 | Up-regulated |
| #6503 | 3049 | 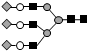 | 27.6 | 17.8 | < 0.0001 | 0.826 | Up-regulated |
| #6513 | 3195 | 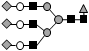 | 16.7 | 5.2 | < 0.0001 | 0.886 | Up-regulated |
| #6523 | 3341 | 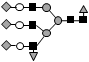 | 0.26 | 0.00 | < 0.0001 | 0.841 | Up-regulated |
| *Tetra-antennary glycans of complex type* | | |  |  |  |  |  |
| #7711 | 3153 | 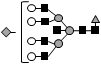 | 0.21 | 0.16 | 0.0005 | 0.724 | Up-regulated |
| #7614 | 3865 | 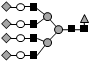 | 0.39 | 0.12 | 0.0003 | 0.757 | Up-regulated |
| #7624 | 4011 | 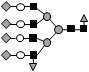 | 0.05 | 0.00 | 0.0004 | 0.822 | Up-regulated |

Note: p-values are from Student’s t-test. Glycans with p < 0.05/61 are shown. # indicates the numbers of monosaccharides, the same as in Figure 2.

^†^Monosaccharide composition: *Rhombus*, sialic acid; *triangle*, fucose; *square*, *N*-acetyl glucosamine; *open circle*, galactose; *closed circle*, mannose.

Abbreviations: AUROC, Area under Receiver Operating Characteristic curve; CD, Crohn’s disease; HLT, healthy volunteers.
